# B-*raf* Alternative Splicing Is Dispensable for Development but Required for Learning and Memory Associated with the Hippocampus in the Adult Mouse

**DOI:** 10.1371/journal.pone.0015272

**Published:** 2010-12-22

**Authors:** Agathe Valluet, Isabelle Hmitou, Sabrina Davis, Sabine Druillennec, Magalie Larcher, Serge Laroche, Alain Eychène

**Affiliations:** 1 Institut Curie, Orsay, France; 2 INSERM U1021, Centre Universitaire, Orsay, France; 3 CNRS UMR 3347, Centre Universitaire, Orsay, France; 4 Université Paris-Sud, Centre de Neurosciences Paris-Sud, Orsay, France; 5 CNRS UMR 8195, Orsay, France; INSERM U862, France

## Abstract

The *B-raf* proto-oncogene exerts essential functions during development and adulthood. It is required for various processes, such as placental development, postnatal nervous system myelination and adult learning and memory. The mouse *B-raf* gene encodes several isoforms resulting from alternative splicing of exons 8b and 9b located in the hinge region upstream of the kinase domain. These alternative sequences modulate the biochemical and biological properties of B-Raf proteins. To gain insight into the physiological importance of *B-raf* alternative splicing, we generated two conditional knockout mice of exons 8b and 9b. Homozygous animals with a constitutive deletion of either exon are healthy and fertile, and survive up to 18 months without any visible abnormalities, demonstrating that alternative splicing is not essential for embryonic development and brain myelination. However, behavioural analyses revealed that expression of exon 9b-containing isoforms is required for B-Raf function in hippocampal-dependent learning and memory. In contrast, mice mutated on exon 8b are not impaired in this function. Interestingly, our results suggest that exon 8b is present only in eutherians and its splicing is differentially regulated among species.

## Introduction

*B-raf* was initially identified as an oncogene transduced into the genome of an acute mitogenic retrovirus able to transform primary cultures of chicken embryonic neuroretina cells [1]. Its human ortholog was simultaneously identified in NIH3T3 cells transfected with Ewing sarcoma DNA [2]. In both cases, the B-Raf protein was truncated in its N-terminus and the kinase domain was fused to foreign sequences leading to its constitutive activation. Since then, *BRAF* turned out to be one of the most frequently mutated protein kinase in human cancers. Thus, *BRAF* oncogenic mutations were detected in about 40-50% of cutaneous melanoma and thyroid papillary carcinoma, 30% of ovarian carcinoma and 15% of colorectal cancers [3], [4], [5].

The vertebrate *raf* gene family contains three members, A-*raf*, B-*raf* and C-*raf* [4], [5], [6]. Knockout studies in mice revealed that Raf proteins display both specific and redundant functions. During early development B-Raf is required for VEGF production by the placenta [7]. Consequently, B-*raf* knockout embryos show impaired development of the labyrinthine layer and die at mid-gestation. In addition, epiblast-restricted knockout mice revealed that B-Raf is dispensable for the development of the embryo proper, which showed normal ERK activation [7]. However, conditional ablation of B*-raf* in neural precursors resulted in severe neurological defects caused by central nervous system (CNS) dysmyelination, leading to death three weeks after birth [8]. These specific B-Raf functions were confirmed by the fact that changing the B-Raf kinase domain with that of A-Raf in knockin experiments was not sufficient to fully rescue the phenotype observed in B-Raf null animals [9]. These findings are likely related to the high levels of B-Raf expression in the nervous system that could not be compensated by other Raf proteins. Indeed, the *B-raf* gene is ubiquitously expressed but displays highest levels of expression in cells and tissues derived from the neuroectoderm, including the neural crest [1], [6], [8], [10]–[12]. In agreement with a critical role in these tissues, gain-of-function germline mutations in the human *BRAF* gene are responsible for cardio-facio-cutaneous syndromes characterized by both anomalies of neural crest-derived structures and mental disorders [12]–[14]. Finally, B-*raf* expression is induced following hippocampal LTP [15] and forebrain-specific knockout of *B-raf* resulted in impairments in hippocampal LTP and forms of hippocampal-dependent learning and memory, including spatial learning and contextual discrimination [16].

The mouse *B-raf* gene encodes several isoforms resulting from alternative splicing of exons 8b and 9b in the hinge region upstream of the kinase domain [17]–[19]. These alternative sequences are specific to *B-raf* as they are not conserved in the other vertebrate *raf* genes or in the unique *raf* ancestor gene in *C. elegans* and *Drosophila*. In agreement with an acquired characteristic specific of *B-raf* following *raf* gene duplication during evolution, *B-raf* sequences corresponding to human exons 3 to 10 are clustered within a single exon in *Drosophila raf*. While 8b-containing transcripts are found in a variety of mouse tissues, 9b-containing transcripts were detected only in tissues of neuroectodermal origin [17], [20]. The presence of exon 8b in the B2-Raf_8b_ isoform and exon 9b in the B3-Raf_9b_ isoform differentially regulates B-Raf by decreasing and increasing kinase and oncogenic activities, respectively [18]. At the molecular level, exons 8b and 9b interfere with the ability of B-Raf N-terminal region to interact with and inhibit the C-terminal kinase domain, thus modulating the auto-inhibition mechanism in an opposite manner [19]. In addition, exons 8b and 9b are flanked by two residues known to regulate B-Raf activity upon phosphorylation. Basal S365 phosphorylation was found higher in the B2-Raf_8b_ isoform than in the B3-Raf_9b_ isoform, resulting in decreased and increased activities, respectively. In contrast, S429 was equally phosphorylated in all B-Raf isoforms, but S429A mutation activated B2-Raf_8b_, whereas it inhibited B3-Raf_9b_ [19]. Taken together, these *in vitro* studies have revealed a subtle and fine-tuned mode of regulation of B-Raf activity. However, while *B-raf* alternative splicing is obvious in avian and mouse tissues [17], [20], B*-raf* splice variants are barely detectable in most established cell lines, suggesting that this mode of regulation is tightly regulated by extracellular signals and/or cell-cell interactions within different tissues, which might be lost in culture conditions. This has precluded the possibility for evaluating potential specific roles of the different B-Raf isoforms, thus far. In the present study, we have generated conditional knockout mice for each B-*raf* alternatively spliced exons. Constitutive ablation of either exon 8b or exon 9b revealed no obvious defects during embryogenesis and adulthood; the animals were viable and fertile, and did not show structural anomalies in their central nervous system. However, behavioural analyses revealed that knockout animals homozygous for the exon 9b mutation were impaired in spatial and object recognition memory, but not in contextual fear conditioning. These results disclose a specific role for exon 9b-containing B-Raf isoforms in certain types of hippocampal-dependent learning and memory.

## Results

### B-*raf* exon 8b is conserved only in eutherians

**Figure 1:**
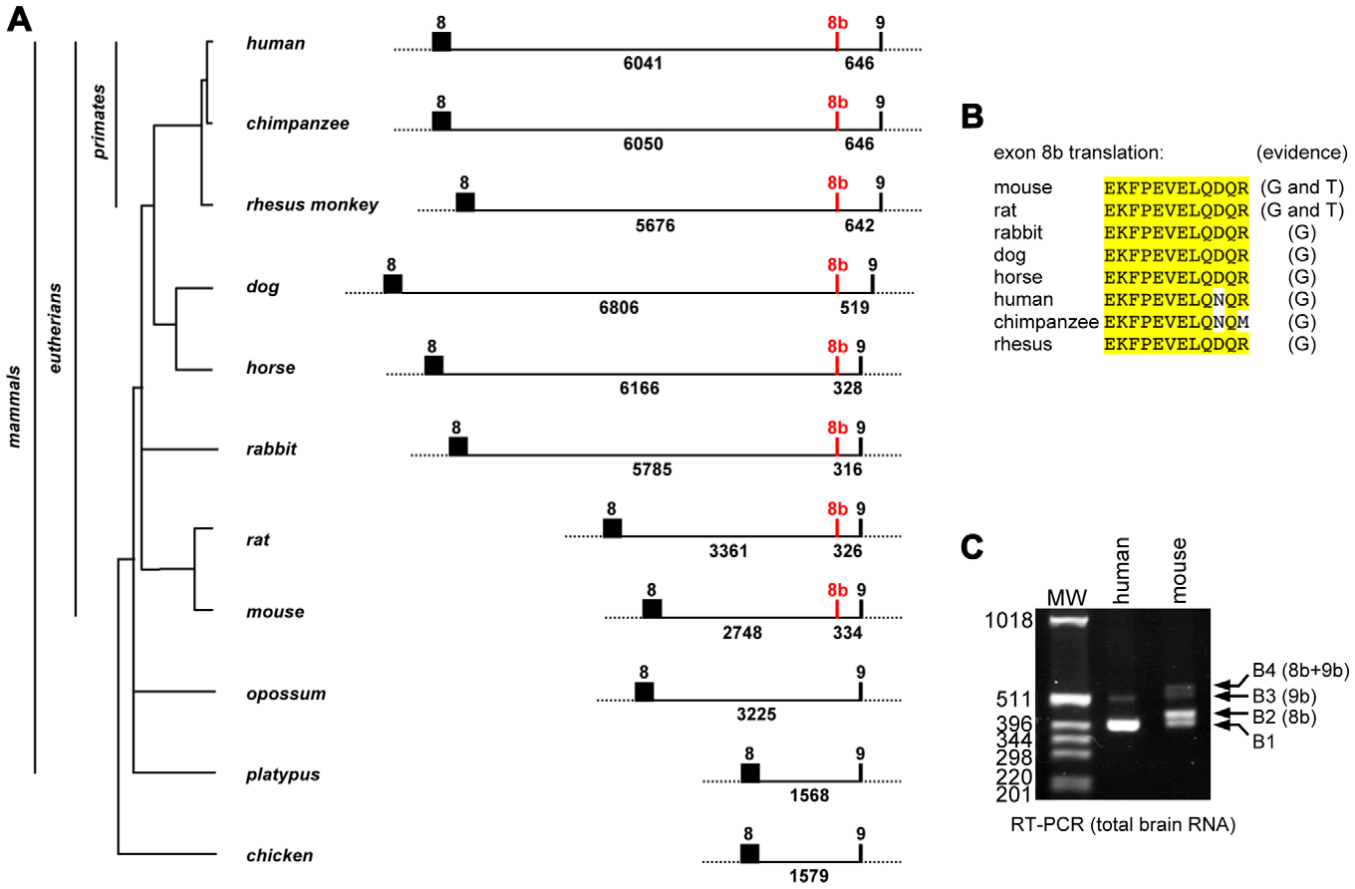
Phylogenetic analysis of B-*raf* exon 8b. (A) Schematic representation of B-*raf* genomic structure between exon 8 and exon 9 in different species. Positioning of intronic and exonic sequences was obtained using ClustalW2 multiple sequence alignment program as described in Suplementary Figure. The size of introns is indicated. Exon 8b sequences are found only in eutherians, but not in other mammals and in chicken. (B) Exon 8b amino-acid sequence conservation in eutherians. Evidence for the presence of B-*raf* exon 8b sequences on genomic DNA (G) or transcripts (T) is indicated on the right. B-*raf* transcripts containing exon 8b have been reported in mouse (This study and Barnier et al., 1995 [17]; cDNA accession number AJ276308) and rat (EST accession number BF543389). (C) Exon 8b is not expressed in human brain. B-Raf isoforms expression was analyzed in human and mouse total brain by RT-PCR as described in Materials and Methods.

We previously reported the high conservation of B-*raf* exon 9b in vertebrates and its presence in transcripts from a wide variety of species, including fish, amphibians, avians and mammals [19]. In contrast, scarce information was available concerning exon 8b. For example, exon 8b-containing B-*raf* transcripts have been reported only in mouse and rat, thus far (Figure 1B and C). A search for B-*raf* exon 8b sequences in available genomic libraries revealed the presence of exon 8b only in eutherians, but not in other mammals such as opossum (marsupialia) and platypus (monotremata), or in avian species (Figure 1A and Figure S1). Despite the presence of exon 8b on genomic DNA from primates, we failed to detect exon 8b-containing transcripts in human brain, testis, heart and various cell lines (Figure 1C and data not shown).

### B-*raf* alternative splicing is dispensable for mouse development

**Figure 2:**
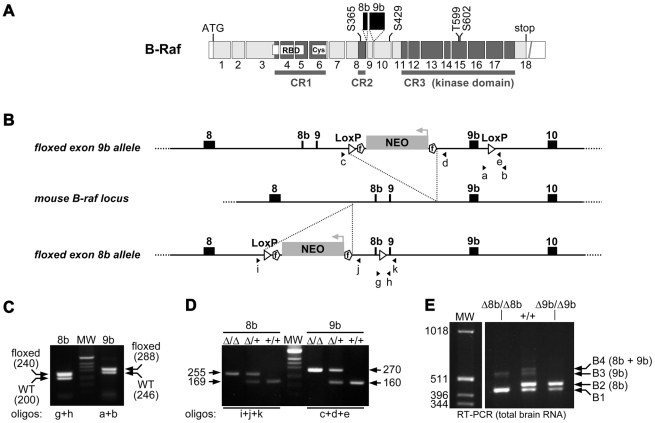
Generation of Δ8b and Δ9b B-*raf* knockout mice. (A) Schematic representation of B-Raf exons structure and conserved regions of the protein (CR1, CR2 and CR3). (B) Targeting strategy and location of primers used for genotyping. (C) PCR analysis on tail biopsies of heterozygous animals carrying a single allele of floxed exon 8b or 9b, as depicted in (B). The size of the amplified fragments is indicated in bp. (D) PCR analysis on tail biopsies after excision of either exon 8b or exon 9b upon crossing to Cre-expressing transgenic lines. The size of the amplified fragments is indicated in bp. (E) RT-PCR amplification of B-Raf isoforms mRNA from adult WT, Δ8b or Δ9b mouse brain. NEO: neomycin gene expression cassette. f: Frt site. WT: wild-type. Δ: deleted allele. Δ8b and Δ9b: allele deleted for exon 8b and 9b, respectively. MW: molecular weight.

To investigate the potential specific functions for B-Raf isoforms during development and adulthood, we generated conditional knockout mice in which either exon 8b or exon 9b was flanked by LoxP sequences (Figure 2B,C). *B-raf^Δ8b/Δ8b^* and *B-raf^Δ9b/Δ9b^* homozygous animals were obtained by crossing to Cre-expressing transgenic lines. Complete conversion of the *B-raf ^f/f^* to *B-raf^Δ/Δ^* alleles was verified both by tail DNA genotyping (Figure 2D) and RT-PCR analyses on brain RNA (Figure 2E). For phenotypic analyses, *B-raf^Δ8b/Δ8b^* and *B-raf^Δ9b/Δ9b^* homozygous animals were compared to wild-type *B-raf^+/+^* and heterozygous *B-raf^Δ8b/+^* or *B-raf^Δ9b/+^* littermates. *B-raf^Δ8b/Δ8b^* and *B-raf^Δ9b/Δ9b^* animals were born at a Mendelian ratio without obvious developmental defects. Adults were viable and fertile, and displayed no defaults in weight (Table 1) or longevity (Table 2), the oldest animals being kept alive for at least 2 years without any detectable anomalies (data not shown). The same observations were made on either mixed 129/Sv-C57Bl/6 or pure 129/Sv background. Unaltered fertility and embryonic development indicated that alternative splicing was not required for B-Raf function in the placenta.

**Table 1:** Mean weight of wild type and knockout animals over time.

	<3 months	3–6 months	6–12 months
+/+	16,98±4,89 (*n* = 11)	24,42±3,10 (*n* = 12)	32,40±6,29 (*n* = 21)
Δ8b/Δ8b	24,05±2,04 (*n* = 8)	28,56±5,00 (*n* = 9)	31,49±5,62 (*n* = 23)
Δ9b/Δ9b	18,59±4,46 (*n* = 8)	26,64±6,74 (*n* = 4)	36,64±6,61 (*n* = 12)

mean weight expressed in g.

**Table 2:** Percentage of surviving wild type and knockout animals over time.

	6 months	12 months	18 months
+/+	92% (*n* = 75)	86% (*n* = 56)	71% (*n* = 38)
Δ8b/Δ8b	100% (*n* = 50)	97% (*n* = 38)	92% (*n* = 24)
Δ9b/Δ9b	94% (*n* = 54)	88% (*n* = 48)	74% (*n* = 42)

**Figure 3:**
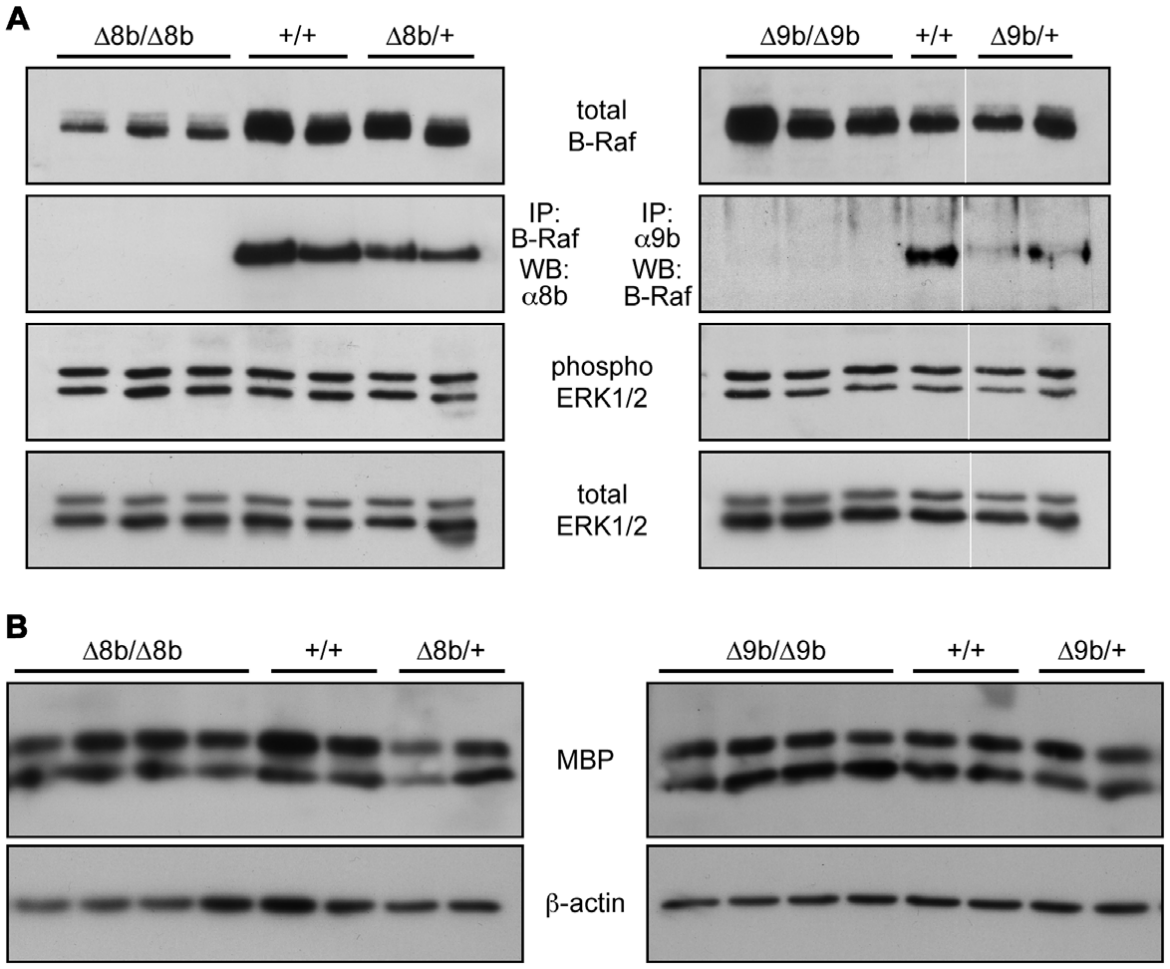
Biochemical analyses on brain from Δ8b and Δ9b B-*raf* knockout mice. (A) B-Raf and phospho-ERK expression. Mouse brain protein extracts were analyzed by direct Western blotting (WB) for the expression of total B-Raf protein, phosphorylated/activated ERK1 and ERK2 (phospho-ERK1/2) and total ERK1 and ERK2 proteins, as indicated. For detection of exon 8b-containing B-Raf isoforms, Western blotting with an anti-exon 8b antibody was performed on immunoprecipitates with the pan-B-Raf antibody. For detection of exon 9b-containing B-Raf isoforms, Western blotting with the pan-B-Raf antibody was performed on immunoprecipitates with an anti-exon 9b antibody. The thin white line on the right panel indicates that the lanes were run on the same gel but were noncontiguous. (B) Myelin basic protein (MBP) expression. Mouse brain protein extracts were analysed by direct Western blotting for the expression of MBP. Western blotting with anti-β-actin antibody was used for loading control.

We further examined the brain of Δ8b and Δ9b knockout mice. Macroscopic analyses of newborn and adult animals did not reveal any gross anomalies. Biochemical analyses of brain protein extracts showed that levels of total and phospho-ERK were similar in knockout and wild type animals (Figure 3A). Conditional ablation of B-Raf in neuronal precursors has been shown to be lethal three weeks after birth and to be associated with reduced myelin basic protein (MBP) production [8]. In contrast, the levels of MBP in the brain of two weeks-old Δ8b and Δ9b knockout mice were similar to those of wild type animals (Figure 3B). Of note, ablation of exon 8b sequences resulted in a significant decrease in the total amount of B-Raf protein in brain, without any effect on ERK phosphorylation and MBP levels. Taken together, these data are consistent with the absence of emergence of neurological defects and growth retardation in Δ8b and Δ9b knockout mice and suggested that alternative splicing is dispensable for B-Raf function in CNS myelination.

### B-*raf* exon 9b is required for certain hippocampal-dependent learning and memory

**Figure 4:**
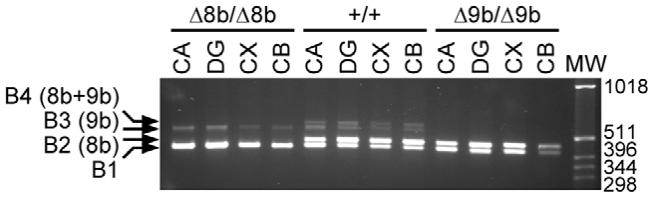
B-*raf* exons 8b and 9b are expressed in the hippocampus of mouse brain. Expression of 8b- and 9b-containing B-*raf* transcripts in the hippocampus (CA: Ammon's horn; DG: dentate gyrus), cortex (CX) and cerebellum (CB), was analyzed by RT-PCR using 5′ and 3′ primers located in exons 8 and 12, respectively. Amplification was performed on dissected regions of adult brain from wild type (WT), Δ8b and Δ9b animals.

**Figure 5:**
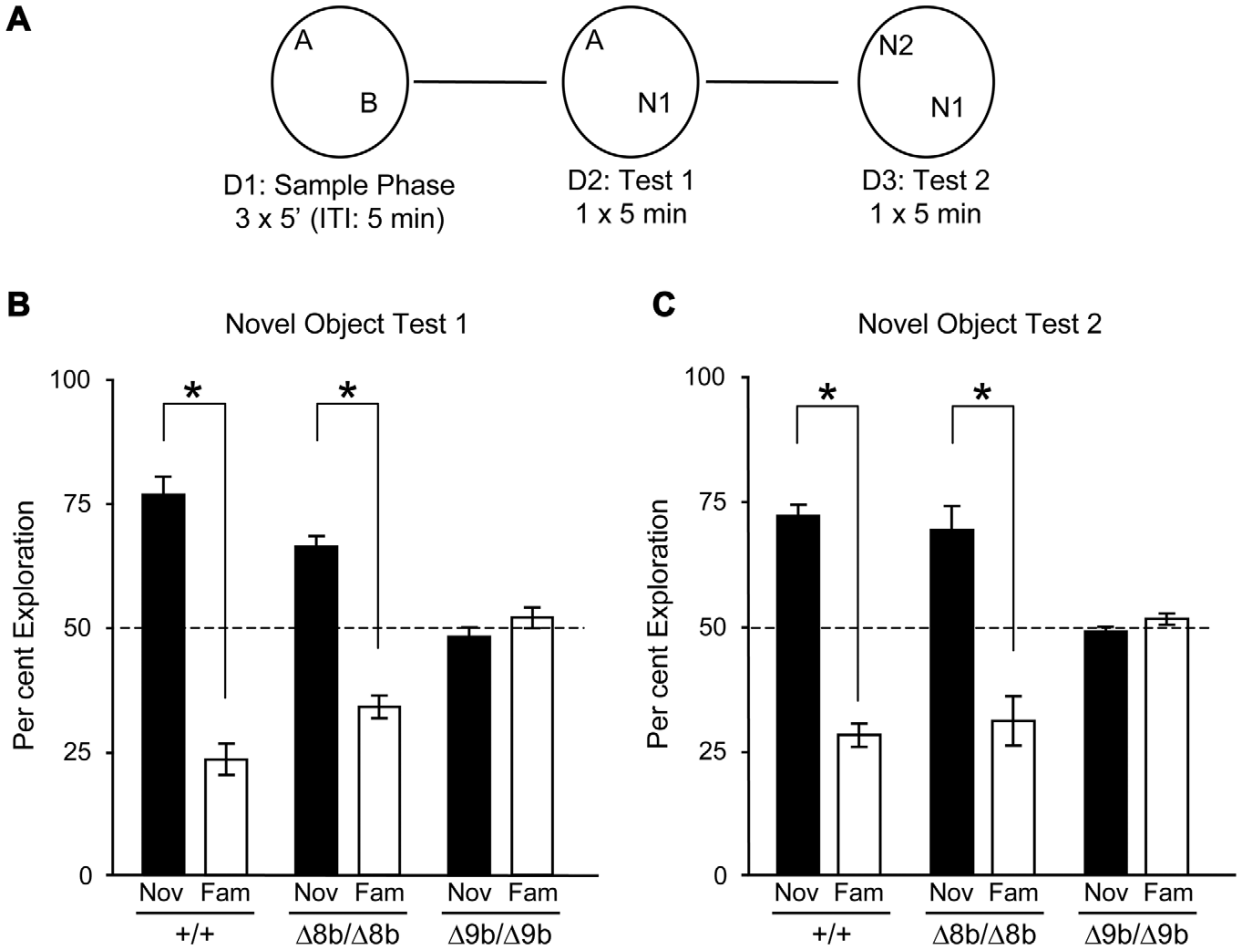
Recognition memory consolidation is impaired in Δ9b B-*raf* knockout mice. (A). Schematic representation of the protocol used for object recognition in a black circular open field. On day 1 mice were given three sessions (5 minutes) with objects they had never observed previously (arbitrarily named A, B). Twenty-four hours later (D2) one of the familiar objects was changed for a novel object (A, N1) and a single trial (5 min) was given to test memory for the objects explored in sample phase. A second test was conducted a 24 hours after the first test where the novel object was now the familiar object (N1) and a novel object replace object A (N2). A single trial of 5 min was given. (B) and (C). Wild type mice (+/+), Δ8b and Δ9b knockout mice were tested on the object recognition task, using the protocol described in panel (A). Representation of the % time spent exploring the novel and familiar objects 24 hours following the acquisition session (panel B, Novel Object Test 1) and 24 hours following the first Novel Object Test (panel C, Novel Object Test 2). In both test sessions, excision of exon 8b had no effect on recognition memory whereas excision of exon 9b did induce impairment compared with wild type mice.

We next examined the possible involvement of B-Raf isoforms in learning and memory. We first verified by RT-PCR that 8b and 9b sequences were expressed in the dentate gyrus and the CA regions of the hippocampus (Figure 4). To assess whether exon 8b and 9b were necessary for learning and memory, knockout mice were tested on an object recognition task in a black circular open field, using the protocol described in Figure 5A. Twenty four hours following the sample phase in the novel object recognition version of the task, statistical analyses showed, as expected, that wild type mice preferentially explored the novel object compared with the familiar object (t(1,12)  = 8.57; p<0.01)(Figure 5B). In a similar manner, *B-raf^Δ8b/Δ8b^* mutant mice also showed preferential exploration of the novel object (t(1,7)  = 7.33; p<0.01). In contrast however, *B-raf^Δ9b/Δ9b^* mutant mice equally explored both familiar and novel objects (t(1,8)  = 1.03; p>0.01), suggesting they did not remember the objects they explored during the sample phase. Analysis of variance conducted on the time spent exploring the novel object confirmed these results by showing a significant difference between groups (F(2,27)  = 28.71; p<0.01). Although post hoc analysis showed the time exploring the novel object was significantly greater in both the wild type and the Δ8b mutants compared with Δ9b mutant mice, the wild type mice showed significantly greater exploration of the novel object compared with the Δ8b mutant mice (see Figure 5B). Twenty-four hours following this test, a second test was given by changing the familiar object in the first test for a novel object (Figure 5A). Similar results were found, where both wild type and Δ8b mutant mice showed significant increase in exploration of the novel object compared with the more familiar object (t(1,12)  = 9.68; p<0.01 and t(1,7)  = 3.89; p<0.01, respectively). In contrast, Δ9b mutant mice explored both objects equally (t(1,8)  = 1.21; p = 0.26)(Figure 5C). As with the first test, ANOVA confirmed a significant group difference in time spent exploring the novel object (F(2,27)  = 19.03; p<0.01) with post hoc analyses showing wild type and Δ8b mutant mice exploring of the novel object more than the Δ9b mutant mice. In this second test however, there was no difference between the time spent exploring the novel object between wild type and Δ8b mutant mice (see Figure 5C).

**Figure 6:**
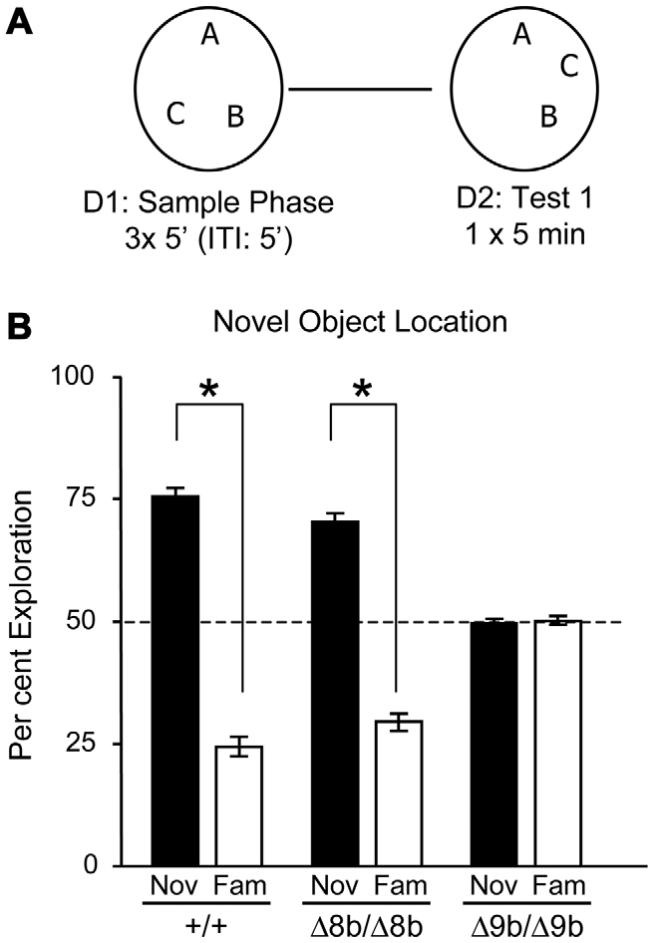
Spatial recognition memory is impaired in Δ9b B-*raf* knockout mice. (A) Schematic representation of the spatial relocation protocol in a black circular open field. The protocol was the same as that for object recognition described in Figure 5, with the exception that three object were used instead of two and the location of one of the objects was changed 24 hours later. (B) Wild type mice (+/+), Δ8b and Δ9b knockout mice were tested on the object relocation task, using the protocol described in panel (A). Representation of the % time spent exploring the displaced object 24 hours following the acquisition phase. Excision of exon 8b had no effect on spatial recognition memory whereas excision of exon 9b did induce impairment compared with wild type mice.

In a spatial version of the task described in Figure 6A, where one of three objects was placed in a novel location, once again we found that both wild type and Δ8b mutant mice showed preferential exploration of the novel location (t(1,12)  = 13.02; p<0.01). In contrast, Δ9b mutant mice did not (t(1,8)  = 0.31; p>0.01) and ANOVA and post hoc analyses confirmed that both wild type and Δ8b mutant mice showed significantly greater exploration of the novel location than did the Δ9b mutant mice (F(2,27)  = 61.51; p<0.01; see Figure 6B). Taken together, these data showed that ablation of exon 9b impairs consolidation of both object and object-place recognition memory, suggesting that this specific function of B-Raf requires alternative splicing of these sequences.

**Figure 7:**
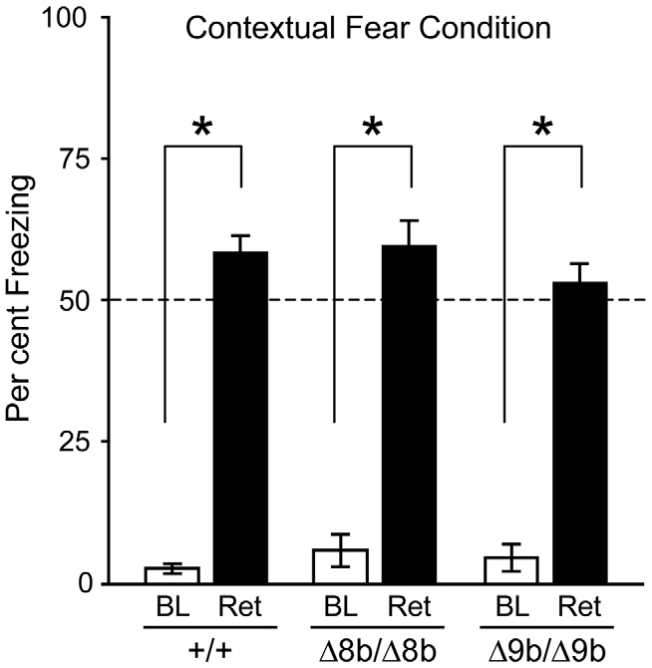
Knockout of B-*raf* exon 8b or 9b does not affect fear conditioning. Δ8b and Δ9b knockout mice were tested for contextual fear conditioning, as described in Materials and Methods. Representation of the % time spent freezing to the context prior to the footshock (BL) and 24 hours later, as a measure of retention of fear memory (Ret). Excision of exon 8b or 9b has no effect on consolidation of fear conditioning.

Complete ablation of all isoforms in forebrain-specific B-*raf* knockout mice selectively impairs contextual discrimination but not contextual fear conditioning [16]. Therefore, to assess the effect of selective ablation of exon 8b and 9b, we tested our Δ8b and Δ9b knockout mice in contextual fear conditioning (Figure 7). As expected all mice showed very little basal freezing during the 2 minute period prior to footshock with no difference in the level of movement between the three groups (F(2,26)  = 1,6; p>0.01). Mice were subsequently returned to the conditioning context 24 hours later, where all exhibited a substantial increase in freezing behaviour measured over two minutes. Comparison of basal freezing with that following a 24 hour delay showed a significant increase in all three groups (wild type: t(1,10)  = 16.37; p<0.01; Δ8b: t(1,7)  = 10.41: p<0.01); Δ9b: t(1,7)  = 10.32; p<0.01), with no significant difference between the three groups in freezing behaviour during the retention test (F(2, 24) = 0.941; p>0.01).

Taken together, these behavioural data show that mutations on exon 8b of the B-*raf* gene has no effect on memory consolidation, at least in the three tasks used here. More importantly, although ablation of exon 9b of B-*raf* does impair memory consolidation, it does so selectively, impairing object and object-place recognition memory but not contextual fear memory.

## Discussion

Among the *raf* genes, B-*raf* is the one displaying the most complex structure. It encodes several protein isoforms through alternative splicing of two exons: 8b and 9b. To evaluate the physiological importance of B-*raf* alternative splicing, we generated conditional knockouts for each alternatively spliced exon. The effect of ablation of either exon was investigated in the three reported essential functions of B-Raf during development and adulthood: placental development, postnatal nervous system myelination and adult learning and memory. Phenotypic analyses of the two knockouts demonstrated that alternative splicing is not essential for the two first functions. Indeed, homozygous animals with constitutive deletion of either exon 8b or exon 9b were viable and fertile and did not show gross anomalies of the nervous system. Thus, none of the animals displayed brain myelination defaults and they survived up to 18 months without any visible abnormalities. To minimize possible compensatory effects, *B-raf^Δ8b/Δ8b^* and *B-raf^Δ9b/Δ9b^* animals were further crossed to *B-raf ^-/+^*;*C-raf ^-/+^* viable compound knockout mice [7]. The resulting *B-raf^Δ8b/-^*;*C-raf ^-/+^* and *B-raf^Δ9b/-^*;*C-raf ^-/+^* animals did not develop obvious phenotype despite the total absence of one *B-raf* and one *C-raf* alleles (data not shown). These results indicate that the absolute level of Raf protein expression is not a critical parameter and that the absence of developmental phenotype in Δ8b and Δ9b knockout mice is not likely to be due to compensatory mechanisms.

We also examined the possible involvement of B-Raf isoforms in hippocampal-dependent learning and memory. Indeed, we showed that 8b and 9b sequences are expressed in both the dentate gyrus and CA regions of the hippocampus. Our behavioural results show a number of important findings. Most notable is the specificity of the role of exon 9b in consolidation of memory. Moreover, the presence of exon 9b-containing B-Raf isoforms is required for selective forms of memory consolidation, where recognition memory is impaired but not fear conditioning. The deficits we observed in Δ9b mutant mice were associated with consolidation and/or retrieval of long-term memory for both the objects and the location of the objects. Furthermore, the deficit in Δ9b mutant mice, but not Δ8b nor the wild type mice, on the second test, which tested recognition memory after a single brief exposure to the objects 24 hours previously, suggested that regardless of whether mice receive single or multiple exposures to the objects, exon 9b-containing B-Raf isoforms play a crucial role in retaining this form of memory. This data is in keeping with a large number of studies, including ours, that established the role of the MAPK/ERK signaling pathway in long-term potentiation (LTP) and both consolidation and reconsolidation of memory associated with hippocampal function [16], [21]–[25]. Downstream targets of B-Raf such as ERK [24], the immediate early gene Egr1 [26] and CREB [22], [27], [28] are all activated and are necessary for the consolidation of recognition memory, suggesting this signaling pathway is a key underlying mechanism necessary for consolidation of long-term memory. Interestingly, CREB activity is also directly regulated by cAMP-dependent protein kinase and, consequently, both cAMP/PKA and ERK pathways converge on CREB in the process [22], [29]–[31]. However, antagonistic effects of the cAMP/PKA pathway on the ERK pathway have been reported. In most of the cells originating from the neuroectoderm including the neural crest, elevated cAMP levels result in ERK activation, through B-Raf activation [6], [10], [32]. Paradoxically, B-Raf protein activity can be inhibited by direct PKA phosphorylation on residues S365 and S429 (Figure 2A)[19], [33]. However, we have shown that exon 9b-containing B-Raf isoforms, which are specifically expressed in cells of neuroectodermal origin, are the most resistant Raf proteins to PKA-mediated inhibition [19]. These observations might provide a possible explanation for our results showing that expression of exon 9b-containing isoforms is required for B-Raf function in hippocampal-dependent learning and memory. To date only a single study has reported the potential role of B-Raf in memory processes and it has been shown that forebrain specific knockout of the entire B-*raf* gene impairs spatial learning and a highly hippocampal-demanding contextual discrimination task, but not contextual or cued fear conditioning [16]. As expected our results showing no deficit in fear conditioning in Δ9b knockout mice are in complete agreement with those reported for the complete knockout of the gene, thereby strengthening the specificity of the role of exon 9b-containing B-Raf isoforms in the consolidation of certain types of hippocampal-dependent memories.

In contrast to B*-raf^Δ9b/Δ9b^* animals, *B-raf^Δ8b/Δ8b^* mice did not show any behavioural deficits. Given that exon 8b-containing transcripts are ubiquitously expressed and represent roughly half of total B-*raf* transcripts in mouse tissues, it is surprising to see no phenotype in Δ8b knockout mice. Indeed, ablation of exon 8b resulted in a significant decrease in total B-Raf protein expression in the brain. However, as mentioned above, *B-raf^Δ8b/-^*;*C-raf ^-/+^* animals did not develop obvious phenotype (data not shown), indicating that decreasing the total level of B-Raf protein in the absence of one *C-raf* allele is not sufficient for revealing a specific function for 8b sequences. In contrast to exon 9b, which is highly conserved in vertebrates and is present in transcripts from a wide variety of species, including fish, amphibians, avians and mammals [19], exon 8b-containing B-*raf* transcripts have been reported only in mouse and rat, thus far (Figure 1B). A search for B-*raf* exon 8b sequences in available genomic libraries revealed the presence of exon 8b only in eutherians, but not in other mammals such as opossum and platypus, or in avian species (Figure 1A and Figure S1). Intriguingly, we failed to detect exon 8b-containing transcripts in human brain, testis, heart and various cell lines (Figure 1C and data not shown). Careful examination of the 8b intron-exon boundaries revealed the presence, in B-*raf* genes from primates, of nucleotide substitutions at critical positions in the consensus sequence of acceptor and donor splice sites (Figure S1). Taken together, these results strongly indicate that alternative splicing of exon 8b is not regulated in a similar manner in primates and in other eutherians, leading to different tissue-specific expression. They also support the notion that exon 8b-containing B-Raf isoforms, which are specific to eutherians, are not critical during development and adulthood, although we cannot exclude highly specialized functions in particular settings, which remain to be identified.

In conclusion, our knockout studies have revealed that alternative splicing of B-*raf* exons 8b and 9b is dispensable for mouse development but the presence of exon 9b-containing B-Raf isoforms is required for learning and memory associated with the hippocampus in the adult mouse.

## Materials and Methods

### Generation of Δ8b and Δ9b B-*raf* knockout mice

Genomic fragments encompassing the mouse B-*raf* locus were isolated from a 129/Sv library. A recombination vector was designed for each alternatively spliced B-*raf* exon. Exon 8b and 9b were flanked by two loxP sites and an frt-PGK-Neo-frt selection cassette was inserted between the 5′ loxP site and the exon. The overall procedure to generate Δ8b and Δ9b knockout mice was essentially as that described by Lecoin *et al*. [34]. Briefly, following electroporation of CK35 ES cells, selected recombinant ES cell clones were screened by Southern blotting using 5′ and 3′ external genomic probes as well as a Neo probe, and injected into C57Bl/6 blastocysts. Floxed allele germline transmission was obtained both on mixed 129/Sv-C57Bl/6 or pure 129/Sv background. For behavioural experiments, the animals were repeatedly backcrossed to obtain pure C57Bl/6 backgrounds. *B-raf^Δ8b/+^* and *B-raf^Δ9b/+^* heterozygotes were obtained by crossing floxed animals either to a PGK-Cre mouse on 129/Sv background or to a ZP3-Cre mouse on C57Bl/6 background [34]–[36].

Genotyping strategies for both floxed and deleted alleles are depicted in Figure 2B. PCR was performed on DNA from tail biopsies using the following primers: (a) 5′- GCGTATGGCTCACATCTGAA-3′; (b) 5′-CATGGTAAAATACTGGACAG-3′; (c) 5′-ATGTGATAGCATATGCCT-3′; (d) 5′-ACAGTCTCAAATGCAATC-3′; (e) 5′-AGGGGCATAAGTCAATAT-3′; (g) 5′-AAGTGCTTCAAACGTTAGTG-3′; (h) 5′-GGTCTCTAATCAAATCCTAC-3′; (i) 5′-CCCTTACACTTAAGTTAAGC-3′; (j) 5′-GGGGAGATTAAAATAGCTCA-3′; (k) 5′-TAAAGTCACTACTTACCTCC-3′.

Animal care and use were approved by the ethics committee of the Curie Institute in compliance with the institutional guidelines. Experimental procedures were conducted in accordance with recommendations of the European Union (86/609/EEC) and the French National Committee (87/848). Alain Eychène's Personal Licence Reference Number: 91-354.

### RT-PCR analyses

RT-PCR analyses of B-Raf isoforms transcripts were performed on total brain or microdissected areas of brain (CA, DG, cortex, cerebellum) from adult WT, Δ8b or Δ9b mice. Total RNA was purified using RNeasy Plus kit (Qiagen). Five µg of RNA were reverse-transcribed (cloned AMV 1st strand cDNA synthesis kit, Invitrogen), amplified with O1/O2 primers and analysed on 2% agarose gels. O1 (5′-TTCCGACCAGCAGATGAAGA-3′) and O2 (5′-TTCAACATTTTCACTGCCAC-3′) primers are located in exons 8 and 12, respectively.

RT-PCR analyses presented in Figure 1 were performed as above, except for the primers: 5′-GCAGATGAAGATCATCGAAA-3′ (located in exon 8) and 5′-TTCAACATTTTCACTGCCAC-3′ (located in exon 12). Both primers recognized human and mouse sequences. Human brain total RNA was purchased from Clontech.

### Western blotting analyses

Dissected mouse brains were lysed in Triton lysis buffer (10 mM Tris pH 7.5, 50 mM NaCl, 1% Triton Tx-100, 1% aprotinin, 1 mM AEBSF, 1 mM sodium orthovanadate, 50 mM NaF, 25 mM β-glycerophosphate) and submitted to serial passages through 18G, 20G, and 25G needles. Insoluble materials were pelleted by centrifugation at 15000× g for 20 min at 4°C. 10–30 µg of protein extracts were used for direct Western blotting. When indicated, immunoprecipitation on 1–3 mg of protein extract was performed prior to Western blotting analysis, as previously described [19]. Samples were resolved by SDS-PAGE, transferred onto PVDF membrane (Millipore) and probed with mouse anti-B-Raf (sc-5284, Santa Cruz Biotechnology), mouse anti-phospho-p42/p44 MAPK (M8159, Sigma), rabbit anti-ERK (sc-93, Santa Cruz Biotechnology), goat anti-MBP (sc-13914, Santa Cruz Biotechnology) and mouse anti-β-actin (Ac15, Sigma) antibodies. For specific detection of 8b- and/or 9b- containing B-Raf isoforms, rabbit polyclonal antibodies were raised against EKFPEVELQ (8b) and APLNQLMRCLRKYQSRTPSPLLHSVP (9b) peptides (Eurogentec). Anti-8b antibody was used at a 1/1000 dilution for Western blotting. Four µl of anti-9b antibody was used for immunoprecipitation. Horseradish peroxidase-conjugated anti-rabbit or anti-mouse antibodies were used as secondary antibodies, and proteins were visualized by ECL (SuperSignal West Dura reagent; Pierce) using either autoradiography or a CCD camera (GeneGnome Bioimaging System; Syngene).

### Behavioural analyses

Behavioural analyses were performed on purified C57Bl/6 backgrounds. At least two independent animal cohorts were used for each test and the experiments were run blind to the genotype.

#### Recognition memory

Mice were tested on an object recognition and an object relocation task in a black circular open field. The protocols are depicted in Figure 5A and 6A, respectively. In the object recognition task two objects were used and in the object relocation task three objects were used. Mice were first habituated to an empty open field for 10 minutes a day for 5 days. The sample phase was comprised of three 5-minute sessions of exploration of objects the mice had never viewed before, with an interval of 5 minutes between the exploration sessions. The following day, either one of the objects was changed for a novel object or the position of one of the three objects was changed in the relocation experiment. In this test session, mice were given a single 5-minute exposure to the objects. In the object recognition task, mice were tested the following day with another novel object replacing the older object. Percent time spent exploring the objects was analysed as a measure of memory consolidation for the objects mice explored during the sample phase and following the first exposure to the novel object.

#### Contextual Fear conditioning

Mice were placed in a context for 2 minutes before delivery of 2 footshocks (Intensity 0.2 mA; duration 2 sec, interval 2 min between shocks) during the acquisition phase. Twenty-four hours later mice were placed back in the same context for 5 minutes. Percent time spent freezing during the first 2 minutes prior to footshock were compared with the first 2 minutes during the retention phase as a measure of memory consolidation of contextual fear.

#### Statistical analyses

Paired Students *t*-test was used to analyse whether there was a significant difference in time spent exploring the novel and familiar objects and the novel and familiar position of the objects within each genotypic group and the wildtype mice. Analysis of Variance (ANOVA) was used to analyse significant differences between genotypic and WT mice in time spent exploring the novel or displaced object. Fisher post hoc analyses identified which groups showed a significant difference.

## Supporting Information

Figure S1 **Comparison of genomic sequences encompassing B-** ***raf*** **exon 8b.** Alignement of B-*raf* genomic sequences of different species from the end of exon 8 to the start of exon 9, using ClustalW2 multiple sequence alignment program (http://www.clustal.org/). Accession numbers: NG_007873 (Homo sapiens); AC187613 (Pan troglodytes (chimpanzee)); NC_007860 (Macaca mulatta (rhesus monkey)); NW_001867414 (Equus caballus (horse)); NW_876260 (Canis familiaris (dog)); NW_003159460 (Oryctolagus cuniculus (rabbit)); NT_039341 (Mus musculus); NW_047690 (Rattus norvegicus); NW_001582020 (Monodelphis domestica (opossum)); NW_001782276 (Ornithorhynchus anatinus (platypus)); NW_001471513 (Gallus gallus (chicken)). The size of introns is indicated in brackets. Black arrows indicate the location of the g->a substitutions in the splice donor and acceptor consensus sequences of exon 8b in primates (human, chimpanzee and rhesus monkey).
(PDF)
