# Supplementary material for: B-raf Alternative Splicing Is Dispensable for Development but Required for Learning and Memory Associated with the Hippocampus in the Adult Mouse
Source: PLoS One. 2010 Dec 22;5(12):e15272. doi: 10.1371/journal.pone.0015272 (PMC3008692; doi:10.1371/journal.pone.0015272)
Supplement: Figure S1 — Comparison of genomic sequences encompassing B- raf exon 8b. Alignement of B-raf genomic sequences of different species from the end of exon 8 to the start of exon 9, using ClustalW2 multiple sequence alignment program (http://www.clustal.org/). Accession numbers: NG_007873 (Homo sapiens); AC187613 (Pan troglodytes (chimpanzee)); NC_007860 (Macaca mulatta (rhesus monkey)); NW_001867414 (Equus caballus (horse)); NW_876260 (Canis familiaris (dog)); NW_003159460 (Oryctolagus cuniculus (rabbit)); NT_039341 (Mus musculus); NW_047690 (Rattus norvegicus); NW_001582020 (Monodelphis domestica (opossum)); NW_001782276 (Ornithorhynchus anatinus (platypus)); NW_001471513 (Gallus gallus (chicken)). The size of introns is indicated in brackets. Black arrows indicate the location of the g->a substitutions in the splice donor and acceptor consensus sequences of exon 8b in primates (human, chimpanzee and rhesus monkey). (PDF) [file pone.0015272.s001.pdf]

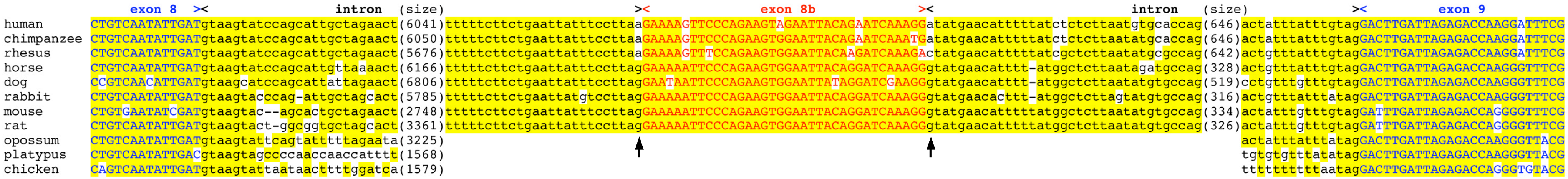

Legend : comparison of genomic sequences encompassing B-*raf* exon 8b.

Alignement of B-*raf* genomic sequences of different species from the end of exon 8 to the start of exon 9, using ClustalW2 multiple sequence alignment program (http://www.clustal.org/). Accession numbers: NG_007873 (Homo sapiens); AC187613 (Pan troglodytes (chimpanzee)); NC_007860 (Macaca mulatta (rhesus monkey)); NW_001867414 (Equus caballus (horse)); NW_876260 (Canis familiaris (dog)); NW_003159460 (Oryctolagus cuniculus (rabbit)); NT_039341 (Mus musculus); NW_047690 (Rattus norvegicus); NW_001582020 (Monodelphis domestica (opossum)); NW_001782276 (Ornithorhynchus anatinus (platypus)); NW_001471513 (Gallus gallus (chicken)). The size of introns is indicated in brackets. Black arrows indicate the location of the g->a substitutions in the splice donor and acceptor consensus sequences of exon 8b in primates (human, chimpanzee and rhesus monkey).

Figure S1: comparison of genomic sequences encompassing B-*raf* exon 8b
